# Pre-diagnostic circulating metabolomics and prostate cancer risk: A systematic review and meta-analysis

**DOI:** 10.1101/2025.02.27.25321444

**Published:** 2025-02-28

**Authors:** Harriett Fuller, Orietta P. Agasaro, Burcu F. Darst

**Affiliations:** 1 Public Health Sciences, Fred Hutchinson Cancer Center, Seattle, Washington, USA; 2 Department of Epidemiology, University of Washington, Seattle, Washington, USA

## Abstract

**Background:**

Metabolomic dysregulation contributes to prostate cancer (PCa) pathogenesis, and studies suggest that circulating metabolites have strong clinical potential to act as biomarkers. However, evidence of circulating metabolite associations has not been quantitively aggregated.

**Methods:**

Systematic searches were performed in PubMed and Embase (October 17^th^, 2024) to identify pre-diagnostic untargeted serum metabolomic studies of PCa risk. After harmonizing metabolite names across studies, restricted maximum likelihood was used to conduct meta-analyses to quantify associations between metabolites and risk of overall PCa, low- to intermediate-risk PCa, high- to very high-risk PCa and lethal PCa, as defined by the NCCN. Statistical significance was defined as FDR-adjusted P<0.05. Enrichment analyses were conducted on significant metabolites to identify biologically relevant pathways. Correlation of effect estimates between PCa outcomes was assessed via Pearson correlation.

**Results:**

We identified 12 untargeted pre-diagnostic circulating metabolomic studies in a systematic review and meta-analyzed associations between up to 408 metabolites with four PCa outcomes. Three, eleven and nineteen metabolites were significantly associated with risk of overall, high/very high-risk and lethal PCa, respectively. Metabolites associated with high/very high-risk PCa were significantly enriched for lipids. Limited evidence of correlation between metabolite effects across outcomes was identified, highlighting potentially unique metabolite drivers of high-risk and lethal PCa. Follow-up analyses found that 13 of the significant metabolites were drug and/or dietary modifiable.

**Conclusions:**

These findings suggest the strong potential for metabolites to inform risk of lethal PCa, which could inform risk-stratified screening strategies and facilitate the identification of targets for PCa prevention.

## Introduction

Globally, prostate cancer (PCa) is the 5^th^ leading cause of cancer mortality and the second most frequently diagnosed cancer in men^1,2^. Although the overall age standardized five-year survival rate of PCa typically ranges from 70–100% (and exceeds 90% in many countries)^3^, survival is considerably lower for individuals with distant metastasis, with survival rates of 34% for distant PCa in the US between 2013–2019 compared to 97% for all cases combined^4^. Despite its high incidence and mortality rates, knowledge of PCa risk factors and pathogenesis remains limited, particularly for more aggressive cancers that have distinctly worse prognosis. Metabolic dysregulation is expected to contribute to PCa pathogenesis, as it accommodates for increased energy demands during cellular malignancy^5,6^. As such, metabolomics provides a promising avenue for better understanding PCa biology and may help elucidate the underlying biological mechanisms that drive tumor development and progression, which could ultimately lead to improved screening strategies and identification of lethal cases.

Although metabolomic epidemiology investigations of PCa risk have been conducted, the replication of metabolites identified to date has been challenging due to limited sample sizes and between-study heterogeneity, particularly regarding sampling strategies, study designs, metabolomic platforms, and data processing and analysis procedures. Accordingly, two previous systematic reviews reported limited reproducibility across circulating metabolomic studies of PCa but highlighted the potential role of amino acids in the diagnosis of PCa and the potential utility of lipids in distinguishing aggressive from non-aggressive PCa at diagnosis^7,8^. However, metabolomic evidence, particularly as it relates to PCa risk prior to disease onset, has not been formally aggregated across studies and evaluated through meta-analyses. With regards to discovery, this is particularly important to pursue across untargeted metabolomic epidemiology investigations, which aim to characterize all metabolites in a sample in an unbiased manner^9–11^.

In this investigation, we conducted a systematic review and meta-analysis of untargeted pre-diagnostic circulating metabolomic studies to quantitatively evaluate current evidence and robustly identify metabolites associated with risk of overall PCa, low- to intermediate-risk PCa, high- to very high-risk PCa and lethal PCa and compare risk profiles across outcomes. Furthermore, we conducted a comprehensive bias assessment to investigate potential causes of heterogeneity present in PCa molecular epidemiological studies.

## Methods

### Search Strategy

This systematic review is registered in the International Prospective Register of Systematic Reviews (PROSPERO; ID CRD42023462809). PubMed and Embase databases were systematically searched by one author (H.F.) for literature published through October 17^th^, 2024, using the following strategy: (‘prostate cancer’ or ‘prostate carcinoma’) AND (metabolite* or metabolomics or metabolomic) AND (serum or blood or plasma or circulating). Mesh indexing, human studies, English language, and original research filters were applied in each database, as applicable. Titles and abstracts were screened in duplicate by two authors (H.F. and O.P.A), and disagreements were mediated by a third author (B.F.D.). Abstract screening was conducted with the Abstrackr tool^12^. Inclusion criteria for the review were published original human research studies that reported on the association between PCa risk and ≥50 circulating metabolites (taken as an indication of an untargeted study) measured prior to prostate cancer diagnosis (i.e., cohort, nested case-control, or case-cohort). Randomized control trials (RCTs) were excluded from the analysis due to the targeted nature of this study design. Given the accessibility of blood samples, which makes blood an ideal target for biomarker development, and the commonality of metabolomic studies based on blood^5^, studies were limited to those conducted in blood samples. Further, studies that did not provide risk estimates for metabolites individually (rather they provided risk estimates for metabolite scores, conducted multivariate analyses, or evaluated prediction models) were also excluded along with studies that did not report metabolites on a continuous scale. Citation lists of included studies were searched until no additional studies were identified.

### Data Extraction and Synthesis

The following variables were extracted from included studies: first author, year, study name, study design, study population/country, case and control sample size, age at sample draw, years between sample draw and diagnosis for cases, prostate cancer definitions, sample type, fasting status, quantification technique, metabolomics platform, number of metabolites identified/investigated, main method of data analysis, covariates included in models, metabolite unit, effect estimates and standard errors. Odds ratios (ORs), risk ratios (RR) and hazard ratios (HRs) were interpreted as relative risks. Study authors were contacted to attempt to obtain missing or incomplete results as needed.

When more than one publication was identified from the same cohort, the publication with the largest number of cases was selected unless it was stated that individuals included in the more recent publication were independent from the previous publication (Supplementary Figure 1). When multiple effect estimates were presented in a study for a given metabolite, effect estimates adjusting for the most covariates were utilized. When effect estimates were only provided for specific strata (i.e., tumor stage, PTEN status, or age categories), the category most comparable to other studies included for the given outcome was selected to minimize heterogeneity. As there were more MS than NMR studies, when MS and NMR estimates were both provided in a single study for a given metabolite, MS estimates were used in meta-analyses. Metabolites were harmonized across studies using COMP IDs (for studies utilizing Metabolon) and manually through the comparison of metabolite names, with alternate names confirmed via the Human Metabolite Database (HMDB)^13^. Metabolites were mapped to distinct biologically relevant pathways based on information provided in identified studies and the HMDB database when discrepancies existed between studies.

### PCa Outcome Definitions

Four PCa outcomes informed by the National Comprehensive Cancer Network (NCCN)^14^ guideline definitions were considered: overall PCa, low- to intermediate-risk PCa, high- to very high-risk PCa and lethal PCa, each compared to PCa-free controls (Supplementary Table 1). The included studies defined overall PCa based on national registries, cancer registries and electronic health records. Due to limited sample sizes, “low- to intermediate-risk PCa” combined low-risk and intermediate-risk PCa, with low-risk defined as patients with Grade Group 1 (Gleason score ≤ 6), cT1-cT2a staging and PSA<10ng/mL and intermediate-risk defined as patients with no high-risk features who had Grade Group 2–3 (Gleason score of 7), PSA of 10–20ng/mL and/or cT2b-cT2c staging. Likewise, “high- to very high-risk PCa” combined high-risk and intermediate-risk PCa and was defined as patients with cancer that had grown outside the prostate (cT3a or above), Grade Group of 4–5 (Gleason score of 8–10) and/or PSA>20ng/mL. Lethal PCa was defined as metastatic PCa or PCa-specific death. Studies defining PCa aggressiveness with definitions that did not match NCCN guidelines were excluded to minimize heterogeneity. Lethal PCa was defined as patients with metastatic disease and/or those whose cause of death was PCa. Each outcome was compared to PCa-free controls.

### Statistical Analysis

Metabolites were meta-analyzed separately for each outcome using a restricted maximum likelihood (REML) approach for metabolites identified in ≥2 studies, as REML reportedly reduces error when included studies have small sample sizes, which is common for metabolomic studies^15,16^. Results obtained from fixed effect meta-analyses were presented when heterogeneity was limited (I^2^≤40%) or if study number was ≤2 and from random effect meta-analyses otherwise. Results were also presented stratified by MS and NMR platforms to assess heterogeneity. Between-platform and between-study heterogeneity was assessed via the I^2^ statistic and a chi-squared test for subgroup differences (Q-value <0.05). An FDR (α=0.05) correction was implemented to account for multiple testing separately for each cancer outcome. Nominal associations were defined as those with an unadjusted P<0.05.

To determine whether metabolites identified were enriched for any biological pathways, enrichment analyses were performed using a one-sided Fisher’s exact hypergeometric test with the R package bc3net^17^. Enrichment analyses were conducted on metabolites with significant and nominally significant (P<0.05) association evidence separately for each PCa outcome, with the reference panel including all metabolite meta-analyzed for a given PCa outcome and the metabolites’ corresponding pathways. The number of observed metabolites for a given pathway was compared to the number of expected metabolites with a given pathway. Significance was defined using an FDR α=0.05 to account for multiple testing separately for each outcome.

We used Pearson correlation to determine how comparable effect estimates were between the different PCa outcomes for overlapping metabolites. This was conducted across all overlapping metabolites, metabolites with an unadjusted P<0.05 in both outcomes, and those with an unadjusted P<0.05 in one or the other outcome. Correlations were not assessed for outcomes with <15 metabolites due to insufficient data.

Results were reported following the Meta-analysis of Observational Studies in Epidemiology (MOOSE) and the Preferred Reporting Items for Systematic reviews and Meta-Analyses (PRISMA) guidelines^18,19^.

### Associations Between Identified Metabolites and Other Cancers and Traits

To evaluate whether any of the significantly associated metabolites were previously associated with cancer or other traits, we searched the HMDB database. Conditions listed under ‘associated disorders and diseases’ and the ‘health effects’ subcategory of physiological effects were extracted. Searches were conducted on December 12^th^, 2024.

### Drug and Diet Metabolite Targets

To explore potential environmental modifiers of the identified significant metabolites, the FooDB database was searched to determine if these metabolites have previously been quantified in foodstuffs, and therefore, if any identified metabolites could potentially be dietary modifiable, using HMDB IDs to search for metabolites. To investigate whether the identified significant metabolites could be drug modifiable, we also searched the DrugBank pharmaco-metabolomics database (https://go.drugbank.com/pharmaco/metabolomics)^20^, utilizing all commonly used synonyms for each metabolite. Searches were conducted on December 12^th^, 2024.

### Risk of Bias (ROB) Assessment

Quality of the included studies was assessed with a published scoring tool^21^, previously utilized in other metabolomic epidemiology systemic reviews^16^, which evaluates six potential sources of bias: study participation, study attrition, exposure assessment, outcome assessment, evaluation of confounders, and the appropriateness of statistical analysis. Each domain was assigned a positive (‘yes’, scoring 1), neutral (‘somewhat’, scoring 0.5) or negative (‘no’, scoring 0) score to indicate whether the study avoided biases in each domain, based upon predefined criteria adopted for this review (Supplementary Table 2). An overall score was summated for each study, and a score ≤3 was taken as an indication of low quality^16^. Question two on study attrition was not considered for nested case-control studies, as information on study attrition details were expected to be reported in the overall cohort study rather than in the nested case-control study. ROB was assessed by two reviewers (H.F and O.P.A).

## Results

### Study Identification

**Figure 1:**
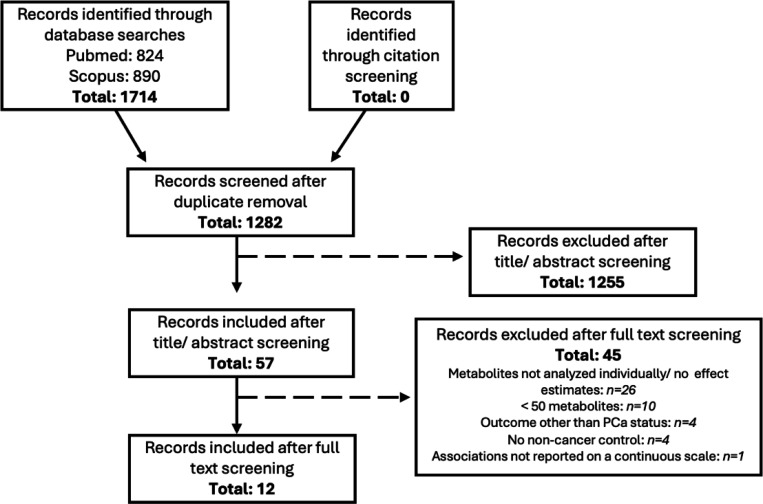
PRISMA flow diagram for systematic review of untargeted metabolomic studies and prostate cancer risk

Systematic searches identified 1,282 records for abstract screening following de-duplication (Figure 1). Abstract screening identified 57 studies eligible for full-text screening. Following full text screening, 12 publications (all of which were nested case-control studies) were identified to be prospective investigations with ≥50 metabolites measured in pre-diagnostic blood samples that reported on individual metabolite associations, making them eligible for inclusion. A total of 15,382 predominantly (>90%) European descent participants, including 7,643 cases and 7,739 controls, were included across 9 cohorts (4 publications from Alpha-Tocopherol, Beta-Carotene Cancer Prevention [ATBC]^22–25^, 2 publications from European Prospective Investigation into Cancer and Nutrition [EPIC]^26,27^, 2 publications from Supplémentation en Vitamines et Minéraux Antioxydants [SU.VI.MAX]^28,29^, and 1 publication each from the Health Professionals Follow-up Study [HPFS]^30^, Northern Sweden Health and Disease Study [NSHDS]^31^, and Cancer Prevention Study-II Nutrition Cohort [CPS-11]^32^; Table 1 and Supplementary Tables 3-4). Ten studies quantified metabolite levels via MS and two used NMR, with one study using both. The average number of metabolites reported across MS studies was 478 (range 67–1,100) and across NMR studies was 208 (range 159–277).

In total, we were able to investigate 595 associations between metabolite biomarkers and any of the four PCa outcomes in meta-analyses, which represented 408 unique metabolite measures (101 amino acids, 8 carbohydrates, 13 cofactors and vitamins, 6 energy related metabolites, 194 lipids, 20 nucleotides, 13 peptides and 53 xenobiotics) (Supplementary Figure 2).

### Overall PCa

**Figure 2:**
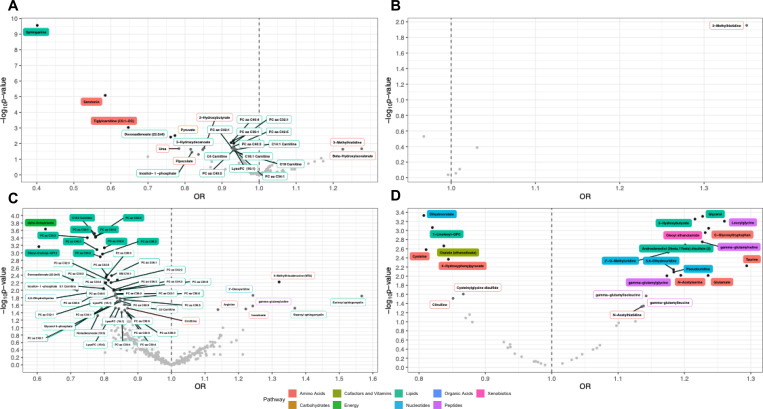
Volcano plots of meta-analysis association results between metabolites and PCa outcomes. Results are shown for A) overall PCa, B) low- to intermediate-risk PCa, C) high- to very high-risk PCa, and D) lethal PCa. Named metabolites were nominally associated (unadjusted P<0.05) with the respective PCa outcome. Shaded labels show FDR significant metabolites. Shading and label outline color represent metabolite class. Light grey point: P≥0.05. Dark grey point: P<0.05. Black point: P<0.01.

**Figure 3:**
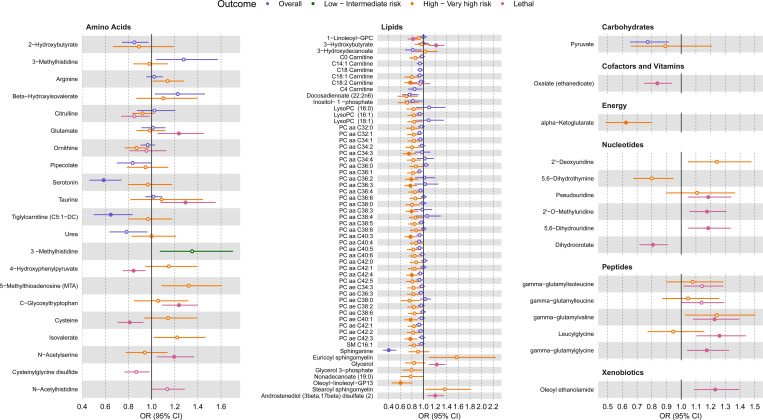
Forest plots of association results for metabolites nominally associated with ≥ 1 PCa outcome. All tested outcomes are shown for each metabolite. Shaded circles indicate FDR significant associations.

Nine studies (6,431 cases, 6,674 controls) reported on the association between 727 metabolite biomarkers and overall PCa risk (Supplementary Table 1). After harmonizing metabolites, 150 metabolites were tested in ≥2 studies and therefore meta-analyzed. After applying an FDR (α=0.05) correction for multiple testing, three metabolites, amino acids serotonin and tiglylcarnitine (C5:1-DC) and the lipid sphinganine, were significantly associated with decreased overall PCa risk (Figure 2 and Supplementary Table 5). In total, 25 metabolites were nominally associated (unadjusted P<0.05) with overall PCa risk (Figures 2–3 and Supplementary Table 5). No metabolite pathway was significantly enriched among metabolites significantly or nominally associated with overall PCa risk (Supplementary Table 6).

Of the 150 metabolites tested, results from 28 were based on both MS and NMR platforms. Evidence of effect heterogeneity by platform was limited, with only four metabolites demonstrating significant heterogeneity (Q-value<0.05), none of which were associated with overall PCa (Supplementary Table 7).

### Low- to Intermediate-risk PCa

Four studies (3,454 cases, 4,505 controls) reported on the association between 186 metabolite biomarkers and risk of low- to intermediate-risk PCa (Supplementary Table 1). After harmonizing metabolites, six metabolites (all amino acids and measured on MS platforms) were tested in ≥2 studies and therefore meta-analyzed. Following an FDR correction, no metabolite was significantly associated with risk of low-intermediate risk PCa (Figure 2 and Supplementary Table 8). One amino acid, 3-methyl histidine, was nominally associated with increased risk of low-intermediate risk of PCa (Figures 2–3, Supplementary Table 8). As all six metabolites were amino acids, enrichment analyses were not conducted.

### High- to Very High-risk PCa

Eight studies (1,470 cases, 6,679 controls) reported on the association between 1,001 metabolite measures and risk of high- to very high-risk PCa (Supplementary Table 1). After harmonizing metabolites, 391 metabolites were included in ≥2 studies and therefore meta-analyzed. Following an FDR correction, 11 metabolites were significantly associated with inverse risk of high- to very high-risk PCa, including 10 lipids, 9 of which were phosphatidylcholines and 1 of which was a carnitine (C18:2 carnitine), and 1 energy metabolite involved in the tricarboxylic acid (TCA) cycle, alpha-ketoglutarate (Figure 2 and Supplementary Table 9). There were 55 metabolites demonstrating nominal association evidence (Figures 2–3 and Supplementary Table 9). Metabolites with significant and nominal association evidence were both enriched for lipids (P_adj_=0.03 and P_adj_=5.71E-09, respectively; Supplementary Table 6).

Of the 391 metabolites tested, results from 23 were based on both NMR and MS platforms. Evidence of effect heterogeneity by platform was identified for one metabolite (glutamine), which was not associated with high- to very high-risk PCa (Supplementary Table 7).

### Lethal PCa

Four studies (1,059 cases, 4,999 controls) reported on the association between 806 metabolites and lethal PCa risk (Supplementary Table 1). After harmonizing metabolites, 48 metabolites (all measured on MS platforms) were included in ≥2 studies and therefore meta-analyzed. Following an FDR correction, 19 metabolites were significantly associated with lethal PCa risk, including positive associations for 14 metabolites (4 amino acids: C-glycosyltryptophan, taurine, glutamate and N-acetylserine; 3 lipids: 3-hydroxybutyrate, glycerol and androstenediol (3beta,17beta) disulfate (2); 3 nucleotides: 2’-O-methyluridine, 5,6-dihydrouridine and pseudouridine; 3 peptides leucylglycine, gamma-glutamylvaline and gamma-glutamylglycine; and 1 xenobiotic: oleoyl ethanolamide) and inverse associations for 5 metabolites (amino acids cysteine and 4-hydroxyphenylpyruvate, cofactor/vitamin oxalate, phospholipid 1-linoleoyl-GPC and nucleotide dihydroorotate) (Figure 2, Supplementary Table 10). There were 24 metabolites demonstrating nominal association evidence (Figures 2–3, Supplementary Table 10). No pathway was significantly enriched among metabolites significantly or nominally associated with lethal PCa risk (Supplementary Table 6).

### Evidence of Between-Study Effect Heterogeneity

**Figure 4:**
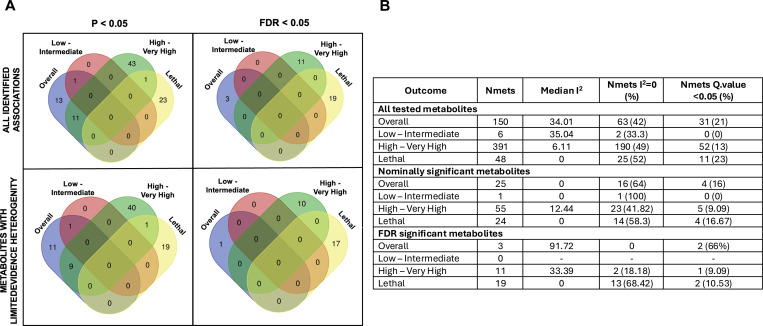
Summary of metabolite effect estimate heterogeneity by PCa outcome and significance threshold. **A**: Overlap of FDR significant and nominally significant metabolites before and after the removal of metabolites where N=2 some evidence of heterogeneity. **B:** Heterogeneity measures across all outcomes. Nmets: Number of Metabolites.

Of the total 595 primary association tests conducted, 280 (47.06%) had an I^2^=0, indicating the absence of between-study heterogeneity. The median I^2^ ranged from 0 for lethal PCa to 35.04 for low- to intermediate-risk PCa (Figure 4). In total, 94 (15.8%) associations had a Q-value<0.05 and an I^2^≥40%, indicating heterogeneity between study estimates. Heterogeneity estimates were largely comparable across metabolite pathways (Supplementary Table 11). The majority of FDR significant metabolites (28/33, 84.85%) and nominally associated metabolites (81/105, 77.14%) had no significant evidence of heterogeneity (defined as Q-value<0.05 and I^2^≥40%) (Figure 4).

### Comparison of Evidence Between Outcomes

**Figure 5:**
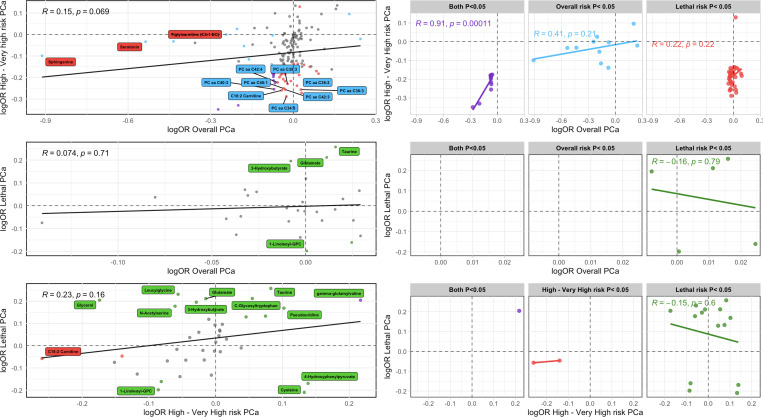
Pearson correlation of metabolite effect estimates between PCa outcomes. **A**: Overall vs high- to very high-risk PCa (N_mets_=143), **B**: Overall vs lethal PCa (N_mets_=27), **C**: High- to very high-risk vs lethal PCa (N_mets_=39). For each comparison, the three panels on the right indicate correlations between metabolites associated with an unadjusted P<0.05 in both traits or in one or the other trait. Purple points indicate results that were associated with both outcomes with an unadjusted P<0.05. Blue points indicate results that were associated with overall PCa with an unadjusted P<0.05. Red points indicate results that were associated with high- to very high-risk PCa with an unadjusted P<0.05. Green points indicate results that were associated with lethal PCa with an unadjusted P<0.05. Grey points indicate results that were associated with both outcomes with P≥ 0.05. Named metabolites were significantly associated (FDR<0.05) with the outcome of the given color. OR plotted on the natural log scale for clarity.

No single metabolite reached the FDR significance threshold for >1 outcome; however, 13 metabolites were nominally associated with >1 outcome (Figure 4), all of which had consistent effect directions across outcomes. To further investigate whether metabolite associations were comparable between PCa outcomes, we assessed correlations between metabolite effect estimates. Due to the limited number of metabolites (n=6) meta-analyzed for low- to intermediate-risk PCa, comparisons were not made for this outcome. Limited evidence of metabolite effects being correlated between outcomes was detected, with the exception of the 11 metabolites nominally (P<0.05) associated with both overall and high- to very high-risk PCa (R=0.91, P<1.1×10^−4^; Figure 5).

### Risk of Bias (ROB)

**Figure 6:**
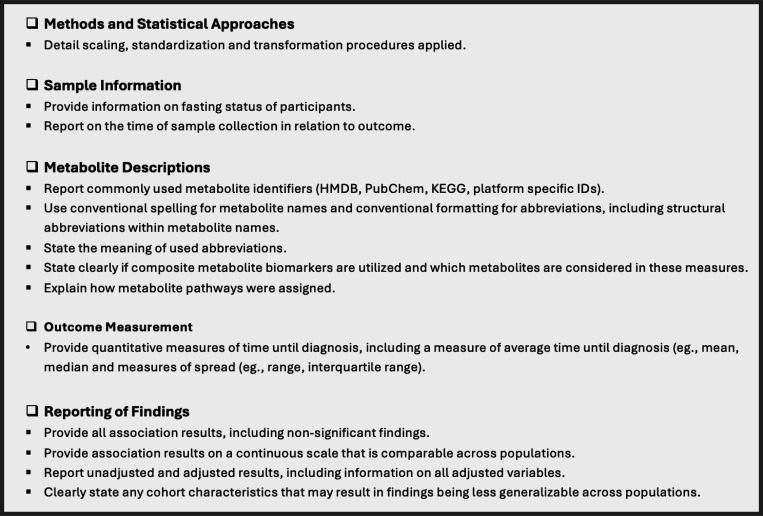
Recommended guidelines to improve reproducibility and facilitate meta-analyses of untargeted metabolomic epidemiology studies.

Our ROB assessment evaluated the quality of the studies included in the meta-analysis and found that bias was most notably identified in the statistical analysis domain, with four studies having low reporting quality (i.e., ROB score ≤3) (Supplementary Table 12). Bias in the statistical assessment domain occurred due to the selective reporting of significant findings in 4/12 studies. Bias in the exposure assessment domain, which was observed to some degree in 6/12 studies, was largely due to some studies not requiring fasting samples (4/12), with one study reporting on m/z values rather than metabolite names, limiting the ability to interpret findings. Bias in the outcome domain occurred in 6/12 studies and was due to reporting on the range/threshold of times between sample collection and PCa diagnosis rather than the median or mean time to diagnosis. Additionally, 7/12 studies were limited to participants that may not represent the general population, such as smokers or healthcare professionals. Participants predominantly represented European ancestries. To address these sources of potential bias, improve metabolite harmonization, and facilitate further metabolomic epidemiology meta-analyses, we have summarized key recommendations that should be considered in future metabolomic epidemiological studies (Figure 6).

### Associations Between Identified Metabolites and Other Cancers and Traits

We also investigated whether the metabolites reported here have been previously associated with other cancers and traits. Of the 33 total metabolites significantly associated with PCa risk, 24 were identified in the HMDB database^13^. In HMDB searches, 17 of these metabolites were previously associated with traits ranging from neurological conditions such as schizophrenia to diabetes and obesity, including 13 metabolites that were associated with ≥1 cancer type (colorectal cancer, pancreatic cancer, lung cancer, breast cancer and leukemia), although none were previously associated with PCa (Supplementary Table 13).

### Drug and Diet Targets

To further explore whether any of the 33 metabolites significantly associated with PCa were modifiable by the environment, the FooDB database was searched to identify potential diet interactions. In total, 21 of the 33 metabolites were identified in FooDB, 8 of which were found to have been previously quantified in foods. Dairy products were the most common type of food item identified, with 5 metabolites previously quantified in dairy products (Supplementary Table 14). An additional 13 of the 33 metabolites are expected to occur in foodstuffs but are yet to be quantified, highlighting the potential utility of dietary intervention in PCa prevention.

Finally, the online DrugBank pharmaco-metabolomics database was searched to determine if any metabolite significantly associated with a PCa outcome was drug modifiable. In total, 2 metabolites significantly associated overall PCa risk were altered by drugs (sphinganine and serotonin), while 7 metabolites significantly associated with lethal PCa risk were altered by drugs (taurine, C-glycosyltryptophan, pseudouridine, 3-hydroxybutyrate, glutamate, glycerol, and cysteine; Supplementary Table 15).

## Discussion

This large-scale systematic review and meta-analysis identified 33 circulating metabolites that were associated with PCa risk, with 3 associated with overall PCa, 11 associated with high- to very high-risk PCa, and 19 associated with lethal PCa risk. Metabolite risk profiles were unique to each PCa outcome, with no metabolites significantly associated with >1 outcome and metabolite effects not correlated between PCa outcomes. This suggests that distinct metabolite drivers may contribute to the development more aggressive PCa, which could have notable implications for tailored risk stratification to discern risk of clinically significant PCa from indolent PCa.

Changes in tumor metabolism that occur with cancer progression are well documented and result in metabolomic heterogeneity between cancer stages, providing an opportunity to identify distinct markers of more aggressive and lethal disease^33^. A metabolomics study of PCa biopsy tissue identified distinct metabolic profiles when comparing tumors with highly aggressive features from less aggressive ones^34^. Seven metabolites significantly differed between tumors with low Ki67 (≤3%; a marker of cellular proliferation) and high PSA (>8ng/mL) from those with high Ki67 and low PSA, including the amino acid taurine and the ratio of glutamate/glutamine measures. Consistent with these findings, we found that taurine was associated with increased risk of lethal PCa but no other PCa outcomes. Further, the long noncoding RNA taurine-upregulated 1 gene (lncRNA *TUG1*), a gene expression regulator that can be upregulated by taurine, has been shown to be upregulated in prostate tumor tissue, with higher lncRNA *TUG1* associated with more aggressive PCa, increased cell proliferation and poorer survival^35^. However, it has also been shown in vitro that taurine may act as a suppressor of metastasis related genes and promote apoptosis.^36^ We also found that glutamate was associated with lethal PCa risk but no other PCa outcomes, consistent with a Mendelian randomization study that found no evidence of an association between glutamate and overall PCa risk^37^. Glutamate can be produced by the glutaminolysis pathway through the breakdown of glutamine and then converted into alpha- ketoglutarate and utilized in the TCA cycle, increasing energy production in cancer cells and facilitating cellular proliferation^38^. Interestingly, we also identified a significant protective effect of alpha-ketoglutarate on high- to very high-risk PCa. Recent work has shown that sine oculis homeobox homolog 1 (SIX1), a newly identified PCa driver, contributes to migration and proliferation by acting as an upstream regulator of the enzyme glutamate-pyruvate transaminase 2 (GPT2), increasing cellular alpha-ketoglutarate production^39^. Future work should further explore the role of glytaminolysis and the TCA cycle in PCa progression and how these cellular processes impact circulating metabolite levels.

Amino acids are expected to contribute to PCa due to their essential role in maintaining redox balance, biosynthesis and homeostatic regulation^40^. Along with taurine and glutamate, we found that amino acids C-glycosyltryptophan and N-acetylserine were associated with increased lethal PCa risk, while cysteine and 4-hydroxyphenolpyruvate were associated with decreased lethal PCa risk. C-glycosyltryptophan has been previously associated with increased all-cause and cardiovascular disease mortality in men from the ATBC Study (which is included in this meta-analysis)^41^. Studies investigating the role of N-acetylserine in PCa development/progression are limited, but higher pre-diagnostic levels of serum N-acetylserine have been associated with increased colorectal cancer risk^42^, and increased urinary levels of N-acetylserine have been observed in patients with endometrial carcinoma^43^. Amino acid 4-hydrophenolpyruvate (also known as 4-hydroxyphenylpruvic acid, (4-HPPA)) is involved in tyrosine catabolism, and 4-HPPA is thought to reduce free radical levels, in turn reducing colon cancer risk^44^. Cysteine is an alpha amino acid involved in protein synthesis. Beyond the studies included in this meta-analysis, observational epidemiological studies investigating the role of cysteine in PCa development/progression and other cancers have been limited and inconclusive.

Dysregulation of lipid metabolism is a characterizing feature of cancer^45^. Accordingly, our investigation demonstrated the involvement of lipids in PCa risk, with 1, 10 and 4 lipids significantly associated with risk of overall, high- to very high-risk and lethal PCa, and with lipids significantly enriched in metabolites nominally and significantly associated with risk of high- to very high-risk PCa. Nine of the 10 lipid metabolites significantly associated with high- to very high-risk PCa were phosphatidylcholines, all of which were associated with reduced risk. Two of these PCs were also nominally associated with reduced risk of overall PCa. PCs are one of the most abundant glycerophospholipids and the most abundant membrane phospholipid, most of which are diacylated—modified by the addition to two acyl groups^46^. PCs act as structural lipids and are precursors to other major membrane phospholipids (sphingomyelins and phosphatidylethanolamines) that can act as secondary messengers. Due to this role in signaling and the generation of lipid mediators, PCs have been associated with both increased and decreased cancer risk^47^, although limited studies have explored the biological mechanisms linking PCs to PCa. Our study also found an additional glycerophospholipid, 1-linoleoyl-GPC, to have a protective association against lethal PCa risk. With the exception of the studies identified in this analysis, no study has investigated the role of 1-linoleoyl-GPC in PCa—however, 1-linoleoyl-GPC was associated with reduced kidney cancer risk in the MetKid consortium^48^.

Three additional lipids, androstenediol (3beta,17beta) disulfate (2), 3-hydroxybutyrate (BHBA) and glycerol, were significantly positively associated with lethal PCa risk. Androstenediols are a type of androgen steroid hormone that promote the growth of both normal and cancerous prostate cells through androgen receptor binding. Androgen deprivation therapy (which decreases androgen levels or blocking androgen receptor binding) is commonly utilized to treat metastatic hormone-sensitive PCa^49^. Unfortunately, resistance to such therapies is known to occur over time, resulting in metastatic hormone-resistant PCa, which is responsible for most PCa deaths^50^. Hence, understanding the role of androgens in PCa development and progression is a major area of research. Androstenediol (3beta,17beta) disulfate (2) is a disulfated androgen, and although the biological role impact of steroid disulfates is not fully understood, the addition of two steroid groups is thought to reduce membrane transferability and receptor binding^51^. Future work is needed to improve our understanding of the role of disulfated androgens in the regulation of androgen signaling pathways, associated with PCa progression through increased cellular proliferation, migration and decreased apoptosis^52^, and to understand if these steroids could be the target of PCa prevention strategies.

BHBA has been associated with increased and decreased cancer risk, likely due the ‘butyrate paradox’ in which some cancers preferentially utilize glucose as fuel and others preferentially oxidize BHBA^53^. BHBA acts as an inhibitor of cell proliferation via histone acetylation; therefore, in cancer types that preferentially oxidize BHBA, lower levels of BHBA are present and cell proliferation is not inhibited^53,54^. Indeed, in a meta-analysis of 1,900 metabolites in blood, urine and tumor samples from 136 cancer cohorts spanning 18 cancers and 21,000 individuals, BHBA was one of the most upregulated blood metabolites across sites^55^. In agreement with our findings, in a targeted multivariate serum metabolomics study, BHBA was found to differentiate PCa cases with and without bone metastasis^56^. Furthermore, BHBA has been shown to increase proliferation and metastasis of colorectal cancer through the regulation of acetyl-CoA acetyltransferase (ACAT1)^57^, the expression of which has been postulated as a cancer therapeutic target and marker for PCa that may distinguish non-aggressive and aggressive disease^58,59^. Glycerol is commonly used as food additive to introduce sweetness to foods. Although considered safe to use in food, our finding aligns with a study reporting that the rate of tumor growth of human PC3 hormone-resistant PCa cells was doubled in mice receiving glycerol compared to control mice receiving a saline solution, which was speculated to be a result of a reduction in oxidative damage^60^.

Four nucleotides were also significantly associated with lethal PCa risk: pseudouridine, 2’-O-methyluridine, 5,6-dihydrouridine and dihydroorotate, the later three of which were associated with increased risk and are related to pyrimidine metabolism. Limited studies have investigated the role of these nucleotides on PCa development; however, dihydroorotate is essential in the de-novo synthesis of pyrimidine and is the substrate for the enzyme dihydroorotate dehydrogenase (DHDOH), the inhibition of which is a potential target for cancer therapy due to the role of pyrimidines in RNA/DNA synthesis and cell proliferation^61^. The nucleotide 2’-deoxyuridine was nominally associated with increased risk of high- to very high-risk PCa but no additional nucleotides were nominally associated with other PCa outcomes. Future work is warranted to better understand the role of pyrimidine nucleotides in lethal PCa and as potential biomarkers of clinically significant PCa.

Finally, three peptides were also associated with an increased risk of lethal PCa: gamma-glutamylvaline, gamma-glutamylglycine and leucylglycine, with gamma-glutamylvaline also nominally associated with increased risk of high- to very high-risk PCa. Beyond the studies included in this investigation, to our knowledge, studies have not examined the role of circulating levels of these peptides in cancer. Future work is warranted to understand how these peptides may act as biomarkers for lethal disease.

Our ROB assessment suggested that the publications included in this review were of good quality; however, key sources of bias were identified. Although bias was typically attributed to features of the primary cohort, including sample collection and the generalizability of studies, the selective reporting of only significant results in 3 (25%) studies could contribute to publication bias and the findings reported in our investigation, potentially introducing false positive and false negative associations. In addition, inconsistent naming of metabolites and the reporting of associations based on spectroscopy measures (e.g., m/z values) could further bias our meta-analysis findings due to the inability to accurately aggregate all reported metabolite associations. Inconsistent adjustment of covariates across studies was observed (Supplementary Table 2) and may have contributed to some evidence of heterogeneity. All identified studies were conducted in predominantly European descent individuals; therefore, results may not be generalizable across populations, which is especially important in PCa given the stark health disparities of this disease, with Black and African American men having significantly higher incidence and mortality rates compared to men from White populations^62,63^. To reduce these burdens, PCa risk factors need to be investigated and understood across population groups.

This is the first study to quantitively aggregate PCa metabolomic investigations to assess the relationship between untargeted circulating metabolites and clinically relevant PCa outcomes, combining the power of previous investigations into a large meta-analysis of ~600 metabolites. As such, this represents the most comprehensive understanding of metabolomic risk of PCa to date. Follow-up analyses of significant metabolites using drug and dietary databases also provides insight into how the identified metabolites could be modified by exogenous factors and offer potential drug targets. However, this study has limitations. Due to the limited number of studies identified and the limited overlap of metabolites between studies, we were unable to investigate potentially confounding factors (e.g., age or follow up time) via meta-regression or formally assess publication bias (e.g., funnel plot inspection or the harbord test). Challenges in metabolite harmonization due to discrepancies in metabolite quantification, identification and annotations, along with the selective reporting of significant findings, have been similarly described in other metabolomic meta-analyses^55,64^ and will be important for the field to address to facilitate evidence synthesis from metabolomic studies and better understand the contribution of metabolites to disease risk. Towards this goal, we propose a set of guidelines for metabolomic epidemiology studies that could mitigate such challenges.

In conclusion, this study has identified a range of metabolites significantly associated with risk of clinically significant PCa. Metabolites associated with risk of high- to very high-risk and lethal PCa, such as glycerophospholipids, amino acids and pyrimidine nucleotides, may have clinical utility as biomarkers for early detection of PCa with poor prognosis. It will be important for future investigations to assess the generalizability of these findings, particularly in high-risk Black and African American populations. Further, it will be important to determine the biological mechanisms driving associations between the metabolites reported here and PCa risk to better understand the potential for these metabolites to serve as biomarkers for PCa prevention, screening, and disease management.

## Supplementary Material

Supplement 1

Supplement 2

## Figures and Tables

**Table 1:** Description of studies included in the systematic review and meta-analysis

Reference	Cohort	Country	Quantification Method	Time from sample collection to diagnosis or PCa death	Number of PCa cases and PCa cases by investigated outcome
Feng et al, 2021^30^	HPFS/PHS	US	LC-MS Broad	Mean time to diagnosis: 5.6–5.9 years	All reported study cases: 488 Overall PCa: 177 Controls: 294
Huang et al, 2017^22^	ATBC	Finland	UHPLC-MS and GC-MS Metabolon	Median time to diagnosis: 10 years (range 1–20 years)	All reported study cases: 137 Low-Intermediate risk PCa: 71 High-Very high risk PCa: 66 Controls: 200
Huang et al, 2019^23^	ATBC	Finland	UHPLC-MS/tandem MS Metabolon	Mean time to PCa death: 18 years	All reported study cases : 523 Lethal PCa: 523 Controls: 523
Lécuyer et al, 2021^29^	SU.VI.MAX	France	Bruker AVANCE III ^1^H NMR^†^	Up to 13 years	All reported study cases: 171 Overall PCa: 171 Low-Intermediate risk PCa: 88 Controls: 171
Lin et al, 2021^28^	SU.VI.MAX	France	UHPLC-HRMS	Mean time to diagnosis: 8.3 years (up to 13 years)	All reported study cases: 146 Overall PCa: 146 Controls: 272
Mondal et al, 2014^25^	ATBC	Finland	UHPLC-MS and GC-MS Metabolon	Range 1–23 years to diagnosis	All reported study cases: 74 Overall PCa: 74 High-Very high risk PCa : 53 Controls: 74
Mondul et al, 2015^24^	ATBC	Finland	UHPLC-MS and GC-MS Metabolon	Range 10–20 years to diagnosis	All reported study cases: 200 Overall PCa: 200 High-Very high risk PCa : 100 Controls: 200
Östman et al, 2022^65^	NSHDS	Sweden	MS	Follow up time >5 years	All reported study cases: 752 Overall PCa: 752 High-Very high risk PCa : 165 Controls: 752
Röhnisch et al, 2020^31^	NSHDS	Sweden	Triple quadrupole MS BIOCRATES NMR	Median time to diagnosis: 10.3 years (range 5–19.9 years)	All reported study cases: 777 Overall PCa: 777 High-Very high risk PCa: 169 Controls: 777
Schmidt et al, 2017^27^	EPIC	European Countries*	Triple quadrupole MS BIOCRATES	39.7% diagnosed <5 years before sample collection 29.3% diagnosed ≥10 years after sample collection	All reported study cases: 1077 Overall PCa: 1077 Low-Intermediate risk PCa: 778 High-Very high risk PCa : 208 Lethal PCa: 127 Controls: 1077
Schmidt et al, 2020^26^	EPIC	European Countries*	Triple quadrupole MS BIOCRATES	Mean time to diagnosis: 9.4 years (4.2 SD)	All reported study cases: 3057 Overall PCa: 3057 Low-Intermediate risk PCa: 2517 High-Very high risk PCa: 580 Lethal PCa: 297 Controls: 3057
Wang et al, 2021^32^	CPS-II	USA	UHPLC-MS/MS Metabolon	44% cases died < 10 years of follow up, 56% died ≥ 10 years of follow up	All reported study cases: 241 High-Very high risk PCa: 129 Lethal PCa: 112 Controls: 342

ATBC: Alpha-Tocopherol, Beta-Carotene Cancer Prevention; CPS-II: Cancer Prevention Study-II Nutrition Cohort; EPIC: European Prospective Investigation into Cancer and Nutrition; GC: Gas Chromatography; LC: Liquid Chromatography; HPFS: Health Professionals Follow-up Study; MS: Mass Spectroscopy; NSHDS: Northern Sweden Health and Disease Study; NHW: Non-Hispanic white; PHS: Physician’s Health Study; PLCO: Prostate, Lung, Colorectal, and Ovarian Cancer Screening Trial; ProtecT: Prostate Testing for Cancer and Treatment; SD: Standard deviation; SU.VI.MAX: Supplémentation en Vitamines et Minéraux Antioxydants; T2D: Type 2 Diabetes; UHPLC-MS: Ultra Performance Liquid Chromatography Mass Spectroscopy. UHPLC-HRMS: Ultra Performance Liquid Chromatography High Resolution Mass Spectroscopy.

* 19 sites across Denmark, Germany, Greece, Italy, the Netherlands, Spain, Sweden, and the UK.

† NOESY1D (NOESY1dgppr sequence) samples and not CPMG (Carr-Purcell-Meiboom-Gill) utilized as larger sample size.

## Data Availability

Underlying data in this article are available in the article and in its online supplementary material.
