# Supplementary material for: Pre-diagnostic circulating metabolomics and prostate cancer risk: A systematic review and meta-analysis
Source: medRxiv. 2025 Feb 28:2025.02.27.25321444. Preprint. [Version 1] doi: 10.1101/2025.02.27.25321444 (PMC11888532; doi:10.1101/2025.02.27.25321444)

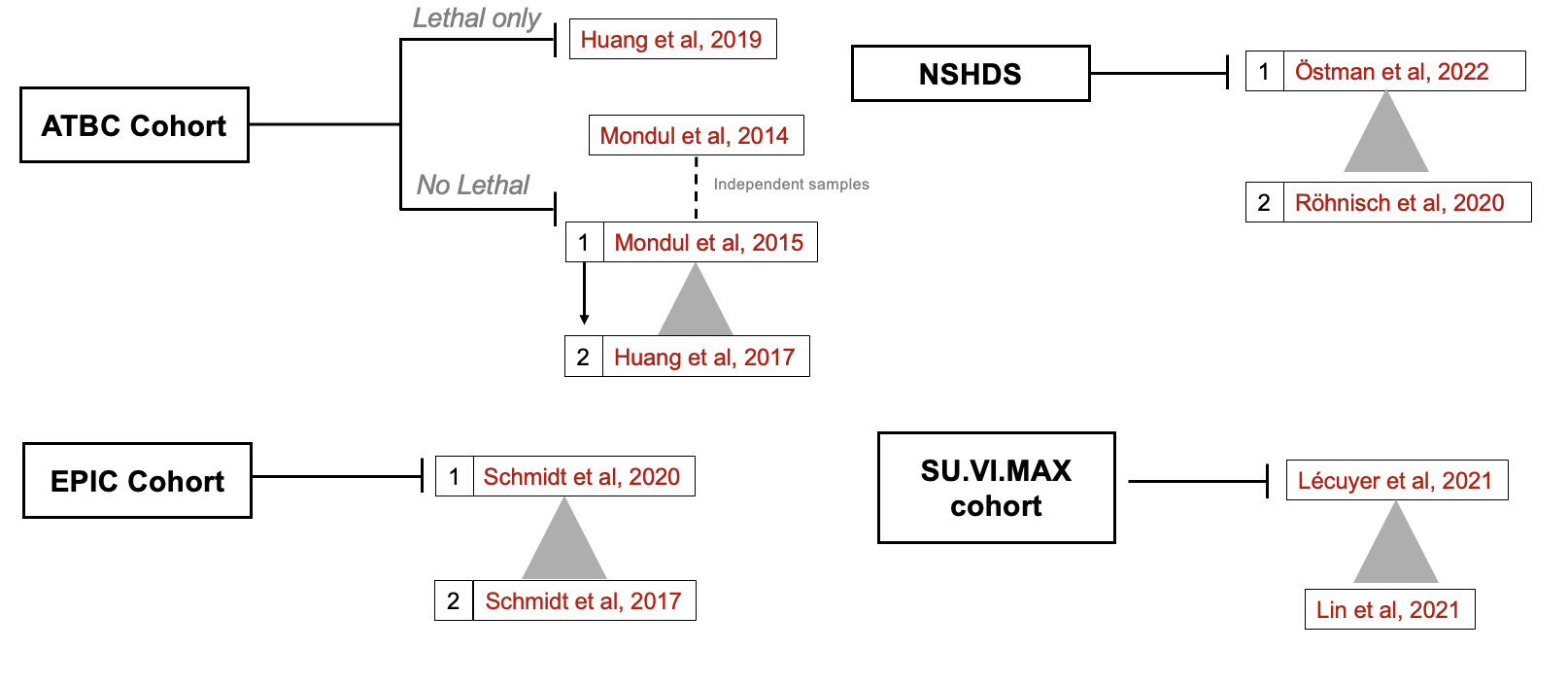
**Supplementary Figure 1: Study prioritization diagram when ≥1 study utilized potentially overlapping samples from the same cohort.** Prioritization was given to the study with the larger number of cases.

**Supplementary Figure 2 Summary of included metabolites. A**: Overlap of investigated metabolites between investigated outcomes. **B**: Total number of metabolites tested for each metabolite pathway. **C**: Number of metabolites tested by pathway and outcome.


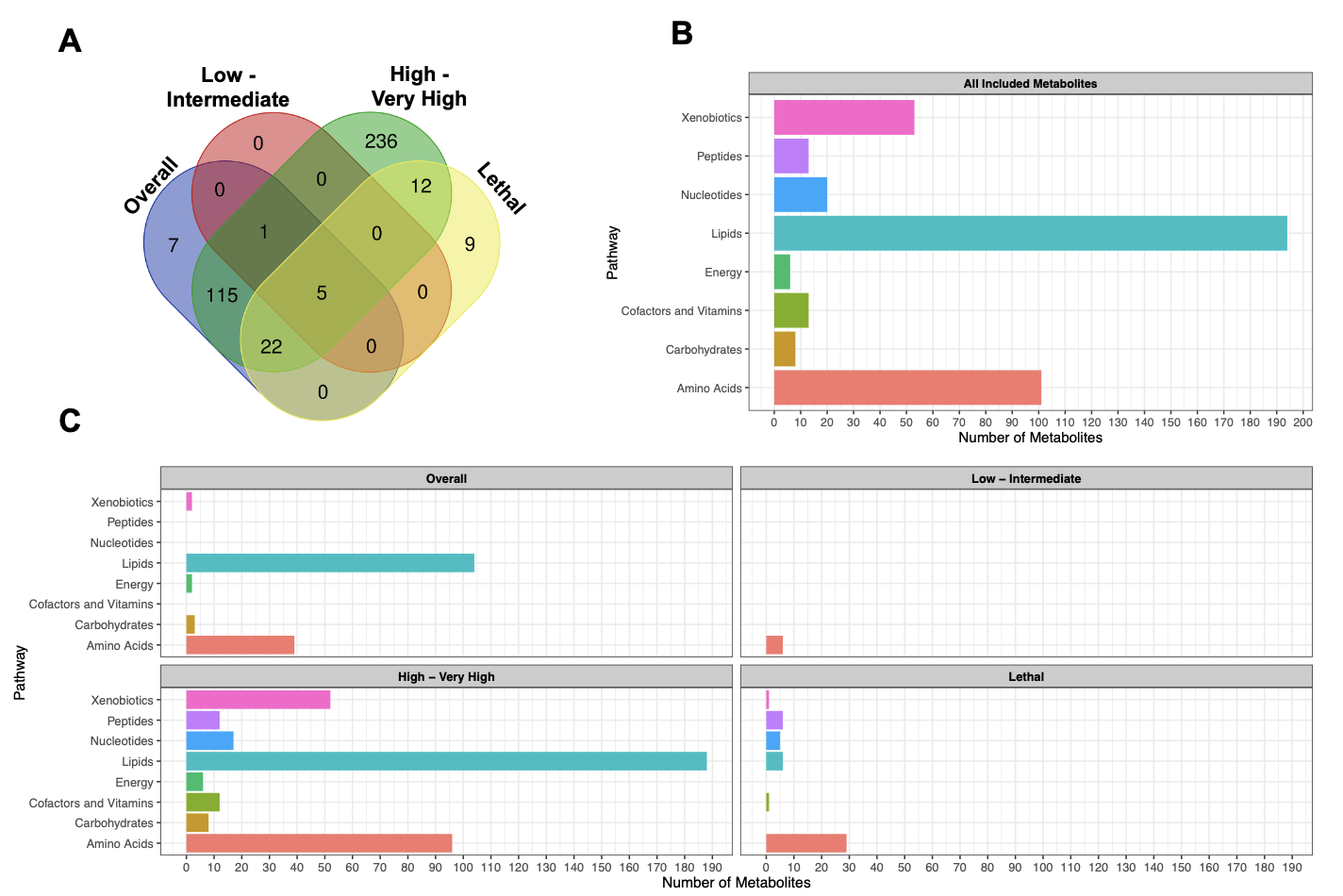

Supplement: Supplement 2 [file media-2.docx]
